# Quantitative Allele-Specific Expression and DNA Methylation Analysis of *H19, IGF2* and *IGF2R* in the Human Placenta across Gestation Reveals *H19* Imprinting Plasticity

**DOI:** 10.1371/journal.pone.0051210

**Published:** 2012-12-05

**Authors:** Sam Buckberry, Tina Bianco-Miotto, Stefan Hiendleder, Claire T. Roberts

**Affiliations:** 1 The Robinson Institute, Research Centre for Reproductive Health, School of Paediatrics and Reproductive Health, The University of Adelaide, Adelaide, South Australia, Australia; 2 The Robinson Institute, Research Centre for Early Origins of Health and Disease, School of Paediatrics and Reproductive Health, The University of Adelaide, Adelaide, South Australia, Australia; 3 JS Davies Epigenetics and Genetics Group, School of Animal and Veterinary Sciences, The University of Adelaide, Adelaide, South Australia, Australia; VU University Medical Center, The Netherlands

## Abstract

Imprinted genes play important roles in placental differentiation, growth and function, with profound effects on fetal development. In humans, *H19* and *IGF2* are imprinted, but imprinting of *IGF2R* remains controversial. The H19 non-coding RNA is a negative regulator of placental growth and altered placental imprinting of *H19-IGF2* has been associated with pregnancy complications such as preeclampsia, which have been attributed to abnormal first trimester placentation. This suggests that changes in imprinting during the first trimester may precede aberrant placental morphogenesis. To better understand imprinting in the human placenta during early gestation, we quantified allele-specific expression for *H19, IGF2* and *IGF2R* in first trimester (6–12 weeks gestation) and term placentae (37–42 weeks gestation) using pyrosequencing. Expression of *IGF2R* was biallelic, with a mean expression ratio of 49∶51 (SD = 0.07), making transient imprinting unlikely. Expression from the repressed *H19* alleles ranged from 1–25% and was higher (*P*<0.001) in first trimester (13.5±8.2%) compared to term (3.4±2.1%) placentae. Surprisingly, despite the known co-regulation of *H19* and *IGF2*, little variation in expression of the repressed *IGF2* alleles was observed (2.7±2.0%). To identify regulatory regions that may be responsible for variation in *H19* allelic expression, we quantified DNA methylation in the *H19-IGF2* imprinting control region and *H19* transcription start site (TSS). Unexpectedly, we found positive correlations (*P*<0.01) between DNA methylation levels and expression of the repressed *H19* allele at 5 CpG’s 2000 bp upstream of the *H19* TSS. Additionally, DNA methylation was significantly higher (*P*<0.05) in first trimester compared with term placentae at 5 CpG’s 39–523 bp upstream of the TSS, but was not correlated with *H19* repressed allele expression. Our data suggest that variation in *H19* imprinting may contribute to early programming of placental phenotype and illustrate the need for quantitative and robust methodologies to further elucidate the role of imprinted genes in normal and pathological placental development.

## Introduction

Genomic imprinting refers to parent-of-origin-dependent allele-specific gene expression. Imprinting affects gene dosage, with the imprinted allele considered repressed and functionally silenced [1], [2]. Imprinting is largely, although not exclusively, observed in eutherian mammals and is thought to have arisen with viviparity and the evolutionary emergence of the chorioallantoic placenta [3], [4]. The prevailing hypothesis on the origin of imprinting is based on paternal-maternal conflict and postulates that paternally expressed genes have been selected to maximize fetal resource acquisition from the mother, while maternally expressed genes have been selected to balance resource allocation to current and future offspring [4]. As imprinted genes appear to facilitate this *tug-of-war* between the maternal and paternal genomes, the conflict hypothesis predicts that imprinted genes are involved in fetal and placental growth and development during pregnancy [2], [4], [5].

Studies using animal models have demonstrated the functional importance of imprinting of *H19, IGF2* and *IGF2R* genes during intrauterine development [6], [7], [8], [9], [10]. Paternally expressed *IGF2* encodes the growth promoting insulin-like growth factor II, a potent mitogen involved in regulating cell proliferation, growth and development. The reciprocally imprinted, maternally expressed *H19* gene is located approximately 130 kb downstream of *IGF2* on human chromosome 11 and encodes a highly expressed, growth regulating, non-coding RNA that shares regulatory elements with *IGF2* [11]. The mechanism by which *H19* interacts with *IGF2* and regulates growth is not fully understood and appears to involve long range interaction of differentially methylated regions and complex loop structures that regulate the activity of parental alleles [12], [13], [14]. More recently, *H19* has been identified as a trans regulator of an imprinted gene network for growth and development [15], apparently through miRNAs processed from the *H19* transcript [11], [16], [17]. The H19 large intergenic non-coding RNA (lincRNA) is highly expressed in extra-embryonic cell lineages and is a developmental reservoir of miR-675 that suppresses placental growth in the mouse [18]. The IGF2 receptor (*IGF2R*) mediates endocytosis and clearance or activation of a variety of ligands involved in the regulation of cell growth and motility, including insulin-like growth factor II [19], [20], [21].

Studies in mice have demonstrated that altered imprinting of *H19, IGF2* and *IGF2R* are associated with placental and fetal growth abnormalities [11], [22], [23], some of which are consistent with data from human studies. For example, (epi)mutations in the *H19-IGF2* region are associated with Silver-Russell and Beckwith-Wiedemann syndromes, which manifest *in utero* in severely growth-restricted and overgrowth phenotypes, respectively [24]. Furthermore, altered epigenetic regulation of the *H19-IGF2* region in human placenta has been associated with pregnancy complications such as preeclampsia, which are preceded by placental pathologies [25], [26]. A significant role in placental development has been established for *H19* and *IGF2* in mouse and human, but knowledge on the role of *IGF2R* in human placental development is limited. The *IGF2R* gene is imprinted in all tissues except brain in mouse, but the majority of human samples indicate non-imprinted biallelic expression [3], [27], [28], [29]. The minority of samples with imprinted or partially imprinted expression suggested developmental stage-specific transient imprinting. However, the developmental role of rare, transient or partial *IGF2R* imprinting in the human placenta [3], [27], [30], [31], [32], [33] remains to be established.

In the human placenta, biallelic expression of imprinted genes, including *H19*, has been observed at higher rates during the first trimester of pregnancy compared to term [25], [34], [35]. Intriguingly, biallelic expression of *H19* in term placentae has been associated with preeclampsia in one study [25], yet subtle variation in *H19* allelic expression in healthy term placentae has also been observed [36]. This limited research on allele-specific expression in the human placenta suggested that imprinting may be dynamic across gestation with potential plasticity in imprinting beyond blastocyst and implantation stages. Although some differences in allele-specific expression of imprinted genes between the first trimester and term human placenta have been reported [34], there appear to be no studies addressing potential changes across the first trimester, a highly dynamic period of placental growth and differentiation. Thus, there is little or no data on temporal variation in imprinting of these genes across gestation, or if imprinting is stable throughout the first trimester and later gestation. In the present study, we quantified the allelic expression ratio for *H19, IGF2* and *IGF2R* and DNA methylation in the *H19-IGF2* imprinting control region across 6–12 weeks of gestation in first trimester placentae and in term placentae between 37–42 weeks of gestation.

## Materials and Methods

### Ethics Statement

Ethics approval was granted by the Children, Youth and Women’s Health Service Research Ethics Committee (REC2249/2/13), the Central Northern Adelaide Health Service Ethics of Human Research Committee (Approval #2005082) and the University of Adelaide Human Research Ethics Committee (H-137-2006). Written informed consent was obtained from all patients.

### Sample Collection

First trimester placental samples ranging from 6–12 weeks of gestation were obtained from elective terminations of pregnancies at the Women’s and Children’s Hospital, South Australia. The consulting physician determined gestational age by observation and the date of the last menstrual period. Placental villous samples were washed in sterile PBS and snap frozen in liquid nitrogen before being stored at −80°C. Term placenta samples were collected from pregnancies classified as being uncomplicated by using the criteria described in [37], and were collected and dissected post-delivery at the Lyell McEwin Health Service, South Australia, and incubated in RNAlater solution (Invitrogen) at 4°C for 24 hours before being stored at −80°C.

### Genotyping

DNA was extracted from placental tissue and parental blood using the Qiagen® DNeasy® blood and tissue kit following the manufacturer’s instructions. DNA concentration was determined using the NanoDrop® ND-1000 Spectrophotometer and diluted to 12.5 ng/µL with nuclease-free water (Mo Bio Laboratories). Isolated DNA from first trimester placental samples was genotyped for *IGF2* rs680, *IGF2R* rs998075 and *IGF2R* rs1570070 single nucleotide polymorphisms (SNPs) by PCR and High Resolution Melt (HRM) analysis (see Methods S1). Term placenta and parental DNA SNP genotypes for *H19* rs217727 and *IGF2* rs680 were determined by multiplex PCR and the Sequenom® MassARRAY® system, using the iPLEX® GOLD single base extension reaction on custom arrays at the Australian Genome Research Facility, Brisbane, Australia.

### Quantification of Allele Specific Expression

Placental samples were thawed and homogenised with 1 mL TRIzol (Invitrogen) per 100 mg tissue. TRIzol (Invitrogen) extraction was performed according to the manufacturer’s guidelines. RNase-free glycogen (Ambion) was added at 25 µg per 1 mL of TRIzol (Invitrogen) to aid in RNA visualisation. RNA samples were DNase treated using the TURBO DNA-free™ kit (Ambion) following the manufacturer’s instructions for rigorous treatment. Following DNase treatment, 2 µL of RNA was subjected to PCR with DNA-specific primers (Table S1). The DNase treatment was determined to be effective if samples showed no amplification after 35 cycles. The concentration of DNase-treated RNA was calculated with the NanoDrop® ND-1000 Spectrophotometer.

First-strand cDNAs were synthesised from 500 ng DNase-treated RNA using the iScript™ cDNA Synthesis Kit (Bio-Rad), following the manufacturer’s instructions. Reverse transcriptase was omitted for negative controls and aliquots of the master mix without added RNA were included in PCR experiments to rule out contamination. Following reverse transcription, cDNA was diluted 1∶10 with nuclease-free water (Mo Bio Laboratories). Aliquots from five cDNA samples were pooled and serially diluted 5-fold for primer validation and PCR optimisation.

PCR primers flanking SNP regions and pyrosequencing primers were designed using the PSQ™ assay design software (Biotage™). Reverse primers featured 5′ biotin modifications and were HPLC purified. All oligonucleotides were synthesised by GeneWorks (Adelaide) and are listed in Table S2. Each sample was pyrosequenced in triplicate, with each replicate generated in an independent PCR cycling run. PCR was performed using 10 µL reactions with 2 µL of cDNA, 5 µL SsoFast EvaGreen Supermix (Bio-Rad) and 300 nM of each primer. Cycling conditions were 2 min enzyme activation at 95°C followed by 40 cycles of 5 sec at 95°C and 20 sec at 60°C. PCR products were sequenced by pyrosequencing using the methods detailed below.

### Quantification of DNA Methylation

DNA for methylation analysis was extracted from placental villous tissue by homogenizing 50–100 mg tissue in 500 µl of TES (10 mM Tris-HCL pH8.0, 1 mM EDTA, 100 mM NaCl), then adding 300 µg Proteinase K and 30 µl of 20% SDS followed by an overnight incubation at 37°C. Then 3 M NaCl was added to precipitate proteins and the supernatant was collected by centrifugation. The DNA was pelleted using 2 volumes of absolute ethanol and washed in 70% ethanol, air dried and resuspended in TE pH 8.0 [38].

Each DNA sample was bisulfite treated in triplicate by EpigenDx (Massachusetts, USA) using 500 ng of DNA and a proprietary bisulfite salt solution followed by incubation for 14 hours at 50°C. Bisulfite treated DNA was purified using Zymogen DNA columns and was eluted with 20 µl of TE pH 8.0, 1 µl of which was used for PCR reactions. The PCR was performed with 0.2 µM of each primer for EpigenDx methylation assays ADS025, ADS596FS and ADS004 with one of the PCR primers being biotinylated for purifying the final PCR product.

### Pyrosequencing

The biotinylated PCR products were bound to Streptavidin Sepharose HP (Amersham Biosciences, Sweden), and the Sepharose beads containing the immobilized PCR product were purified, washed and denatured using a 0.2 M NaOH solution and rewashed all using the Pyrosequencing Vacuum Prep Tool (Qiagen) as recommended by the manufacturer. Then 0.2 µM pyrosequencing primer was annealed to the purified single-stranded PCR product. 10 µl of the PCR products were sequenced using the PSQ96 HS System (Biotage AB) following the manufacturer’s instructions at EpigenDX Genome and Epigenome Research Facility (Massachusetts, USA). The status of each locus was analyzed individually using QCpG software (Qiagen).

### Statistical Analysis

Repressed allele expression differences between gestational age classes for each gene were analyzed using one-way analysis of variance (ANOVA). Differences between first trimester (6–12 weeks of gestation) and term (37–42 weeks of gestation) samples were analyzed using the t-test. Differences in allelic expression measured at two loci in the one sample were analyzed using the paired t-test. The relationship between repressed allele expression from two genes in the same sample was tested by calculating the Pearson’s bivariate correlation coefficient. Differences in levels of DNA methylation between first trimester and term samples were tested for each individual CpG site and for each region using independent t-tests. The relationship between repressed allele expression and mean DNA methylation for each region and CpG site was tested using Pearson’s correlation. Results were considered significant at *P*<0.05. All statistical analyses were performed using GraphPad PRISM 5.0.

## Results

### Quantification of Allele-specific Gene Expression in the Human Placenta

DNA samples from placental tissue was genotyped for SNPs *H19* rs217727, *IGF2* rs680, *IGF2R* rs998075 and *IGF2*R rs1970070 to identify heterozygous individuals. Sixty-nine samples in total were heterozygous for at least one of the tested candidate SNPs. The number of heterozygotes identified for each gestational age class is summarized in Table 1. As parental DNA corresponding to term placenta samples was available for 28 cases, we genotyped maternal and paternal DNA for *H19* and *IGF2* polymorphisms to determine the parental origin of expressed alleles. In all cases with sufficient parental genotype information, *H19* was maternally expressed (n = 11) and *IGF2* (n = 9) was paternally expressed, as expected (Table S3).

**Table 1:** Number of informative heterozygous samples for *H19*, *IGF2* and *IGF2*R for each gestational age class.

	Number of Heterozygotes		
Gestational Age (weeks)	*H19*	*IGF2*	*IGF2R*
6	1	1	1
7–8	6	5	14
9–10	4	6	8
11–12	2	2	1
37	1	0	NA
38	0	0	NA
39	7	5	NA
40	5	8	NA
41	5	6	NA
42	0	1	NA
**Total**	**31**	**34**	**24**

First trimester samples range from 6–12 weeks, term samples range from 37–42 weeks.

**Figure 1:**
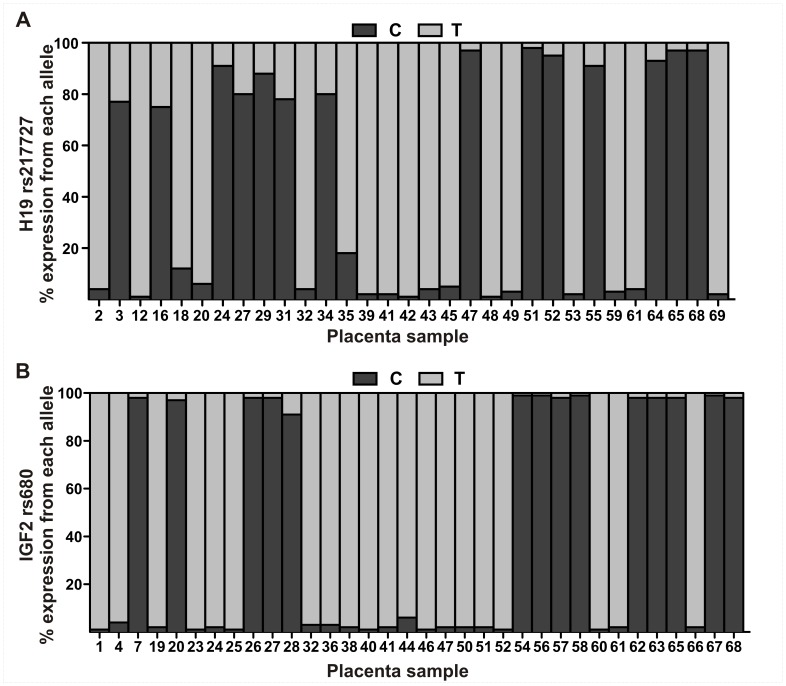
Relative expression from *H19* and *IGF2* alleles in the human placenta. Shaded bars for (A) *H19* and (B) *IGF2* represent the proportion of expression (%) from each allele. Samples 1–38 are from first trimester (6–12 weeks of gestation) placentae and samples 39–69 are from term (37–42 weeks of gestation) placentae.

**Figure 2:**
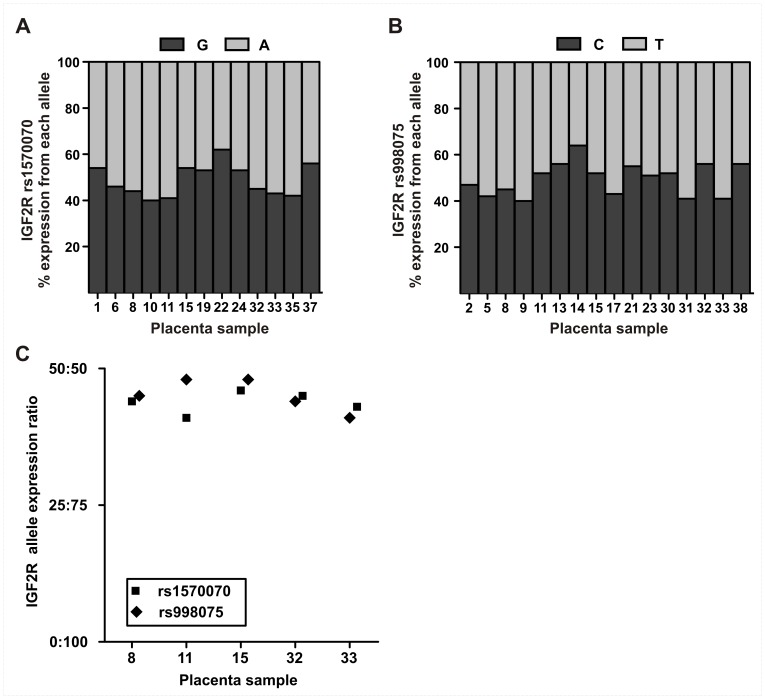
Relative expression from *IGF2R* alleles in the human placenta. Shaded bars for (A) *IGF2R* rs1570070 and (B) *IGF2R* rs998075 represent the proportion of expression (%) in first trimester placentae. (C) Allelic expression ratios for *IGF2R* measured two SNP loci in the same sample. These paired samples indicate both SNP loci are equivalent (paired *t*-test, *P* = 0.42) for evaluating *IGF2R* allele-specific expression.

**Figure 3:**
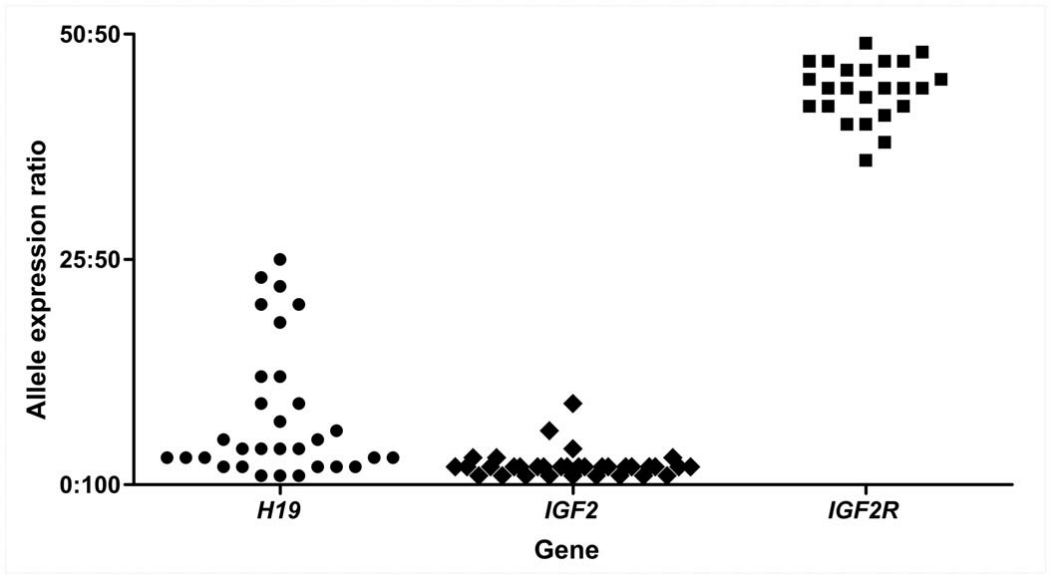
Allelic expression ratios for *H19, IGF2* and *IGF2R* in the human placenta. The 50∶50 ratio represents equal expression from both alleles and 0∶100 ratio represents expression exclusively from one allele. Each point on the graph represents the allelic expression ratio measured in an individual placental sample. *H19* and *IGF2* samples are from first trimester and term placentae, *IGF2R* samples are all from first trimester placentae.

Relative expression from each *H19*, *IGF2* and *IGF2R* allele was quantified in placenta samples by pyrosequencing of SNP loci. Relative allelic expression levels for *H19*, *IGF2* and *IGF2R* in first trimester and term placenta samples are presented in Figures 1 and 2 with each gene showing a unique allele expression profile. Technical replicates obtained from independent PCR reactions showed average standard deviations (SD) of 0.44% for *H19* rs217727, 1.02% for *IGF2* rs680, 3.14% for *IGF2R* rs998075 and 3.84% for *IGF2R* rs1570070, respectively, indicating robust assays with negligible inter-PCR variation. The greater standard deviation for *IGF2R* replicates is likely due to the higher PCR quantification cycle (Cq) required for data acquisition as compared with *H19* and *IGF2* (data not shown). The ratio of repressed allele to predominantly expressed allele is depicted in Figure 3, where a 0∶100 ratio represents no expression from the repressed allele and a 50∶50 ratio represents balanced, i.e., bialleleic, non-imprinted expression. Across first trimester gestational age classes, expression from the *H19* repressed allele shows notable inter-individual variation in contrast to the almost uniform monoallelic expression observed for *IGF2* (Figure 3). *IGF2R* allele-specific expression in first trimester placenta samples showed balanced expression, with some samples potentially showing a slight allelic bias (Figures 2, 3).

### Biallelic Expression of *IGF2R* in the First Trimester Placenta

Allelic expression ratios for *IGF2R* in first trimester placenta was measured at two SNP loci (rs998075 n = 16 and rs1570070 n = 13). Five samples were heterozygous for both SNPs, and no significant difference (paired *t*-test, *P* = 0.42) was detected between the expression ratios for the two SNP loci, indicating that both polymorphisms were equivalent in quantifying allele-specific expression (Figure 2C). All heterozygous *IGF2R* samples were therefore combined for analyses, and, when expression was quantified at both loci in one sample, the average allelic ratio of the two loci was used. The results clearly show biallelic *IGF2R* expression in all first trimester placental samples assessed (Figures 2A, B), with a mean allele expression ratio of 49∶51 at both SNP loci (rs998075 SD = 7.1%, rs1570070 SD = 6.9%) with expression ratios ranging from 36∶64 to 49∶51 (Figure 3). These SNP based *IGF2R* pyrosequencing results provide no evidence for *IGF2R* imprinted expression in the first trimester placenta and thus confirm the non-imprinted status of *IGF2R* throughout gestation.

### Increased Expression from the *H19* Repressed Allele is Higher in First Trimester Placenta

**Figure 4:**
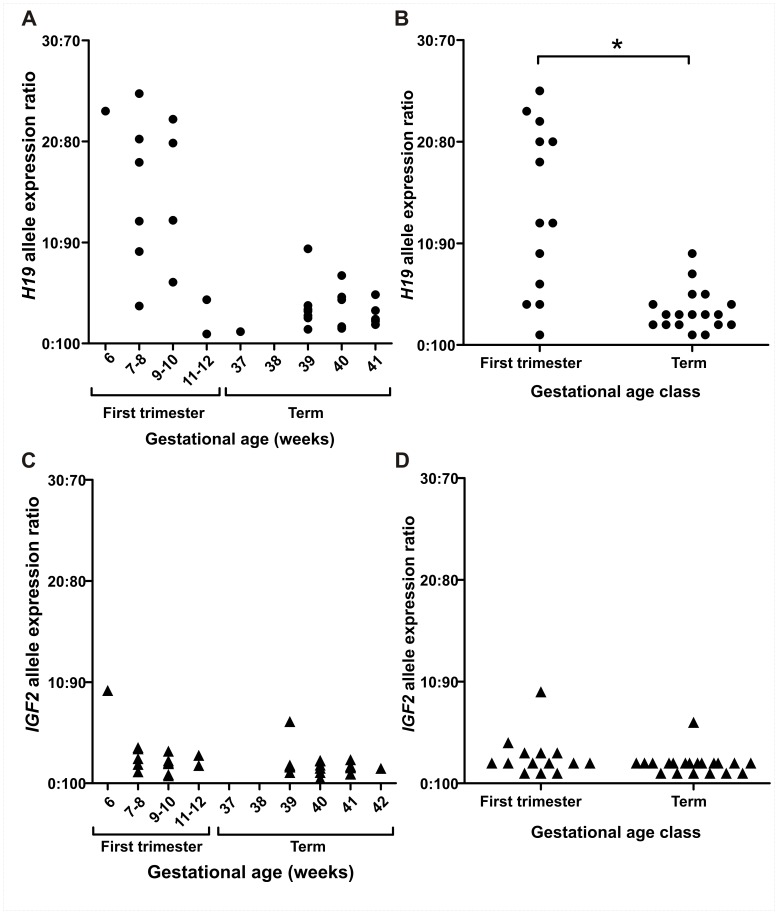
Ratio of expression from each allele in human first trimester and term placentae measured by pyrosequencing. Each point on the graph represents the allelic expression ratio observed in an individual placental sample. (**A**) *H19* allelic expression ratio for each gestational age class. (**B**) Expression from the *H19* repressed allele is significantly higher (*P<0.001) in first trimester placental samples. (**C-D**) *IGF2* allelic expression ratios are similar for each gestational age class (**C**) with no significant difference (**D**) between first trimester and term. First trimester samples are 6–12 weeks of gestation term samples are 37–42 weeks of gestation.

Expression from the *H19* repressed allele was quantified in 13 first trimester placenta samples obtained at 6–12 weeks of gestation (Figure 1A). Mean expression from the repressed allele was 13.5% (SD±8.2; Figure 1A) and ranged from 0.9–24.7% (Figure 3). Expression of the *H19* repressed allele appeared to decrease with gestational age in the first trimester samples (Figure 4A), but we found no significant differences between first trimester gestational age classes. To further test the hypothesis that expression from the repressed *H19* allele decreases across gestation, we then quantified allelic expression in term placenta samples obtained between 37–42 weeks of gestation (n = 18). Expression from the repressed *H19* allele at term was significantly lower (*P*<0.001) than the level of expression observed in first trimester placenta samples (Figure 4B and Table 2).

**Table 2:** Relative levels of repressed allele expression in human first trimester and term placentae.

	% Repressed allele expression		
	First trimester	Term	P value
***H19***	13.5±8.3	3.4±2.0	<0.0001
***IGF2***	2.6±2.0	1.9±1.1	0.1734
***IGF2R***	43.8±3.2	ND	–

ND = Not Determined.

Expression from the *IGF2* repressed allele contributed on average 2.7% (SD 2.1%, n = 34) to total *IGF2* transcript in placenta samples (Figure 1B). No significant differences in expression from the *IGF2* repressed allele were observed between first trimester gestational age classes (ANOVA *P*>0.05) or between first trimester and term (Table 2, Figures 4C, D) placentae. These results show that imprinted *IGF2* expression is tightly regulated and stable across gestation.

**Figure 5:**
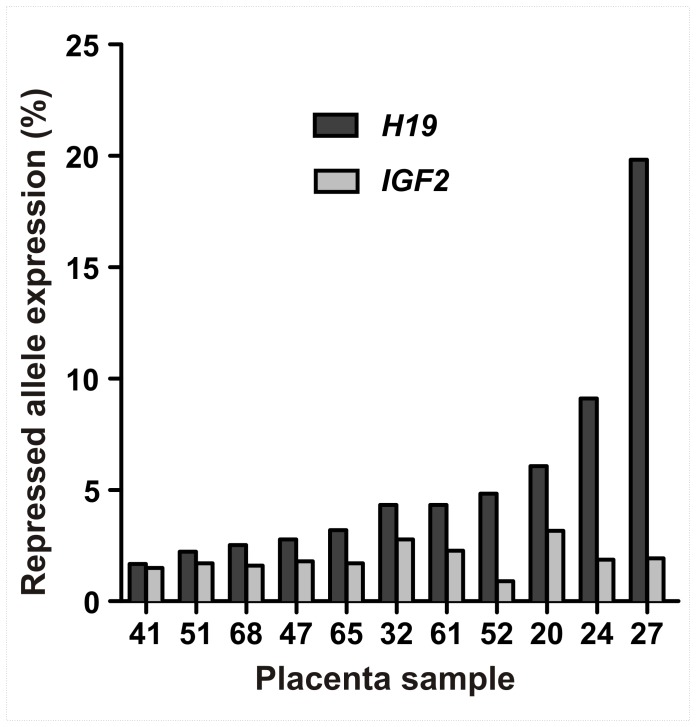
Relative level of expression from repressed alleles in samples heterozygous for both *H19* rs217727 and *IGF2* rs680. Graph shows increased expression from the *H19* repressed allele is not correlated (*P* = 0.09, r = 0.54) with expression from the *IGF2* repressed allele.

Eleven placenta samples were heterozygous for both *H19* and *IGF2* polymorphisms, which allowed us to test for a correlation in repressed allele expression for these adjacent co-regulated imprinted genes. We found that expression from the *H19* repressed allele was not correlated (*P* = 0.88, r = 0.54) with expression from the *IGF2* repressed allele (Figure 5).

### Locus-specific DNA Methylation Differences in the *H19*-*IGF2* Region between First Trimester and Term Placentae

**Figure 6:**
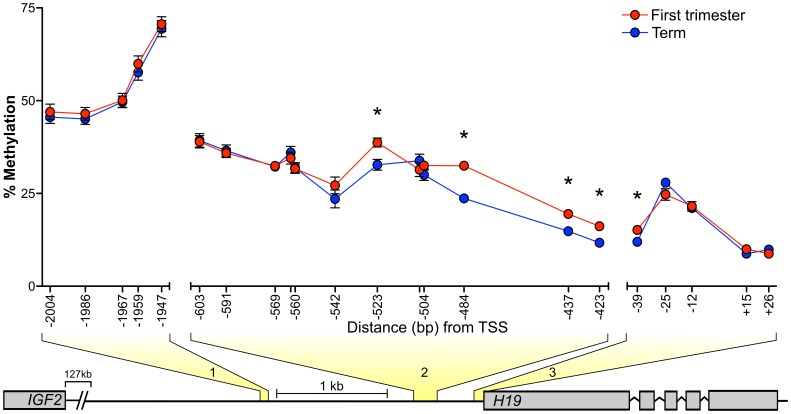
Placental methylation levels in regions upstream and covering the *H19* transcription start site (TSS). Each genomic region where DNA methylation was measured is highlighted in yellow and numbered 1–3. DNA methylation levels for individual CpG loci are shown for first trimester (red circles, n = 13) and term (blue circles, n = 18) placentae. Distance (bp) of cytosine nucleotide from *H19* TSS is represented on x-axis. Each data point represents the mean methylation level for the gestational age class. * Indicates a significant difference in methylation levels between first trimester and term placentae at individual CpG sites. Error bars represent SEM and when not present SEM was too low to depict on the graph. The schematic representation below the graph highlights the regions between *H19* and *IGF2* where bisulfite DNA pyrosequencing was performed. Region 1 covers 5 CpG sites (Chr11∶2021011–2021070), region 2 covers 12 CpG sites (Chr11∶2021011–2021070) and region 3 covers 5 CpG sites (Chr11∶2019079–2019145). Genomic coordinates refer to reference assembly GRCh37/hg19.

To investigate if DNA methylation levels at specific CpG’s are correlated with *H19* repressed allele expression, we quantified methylation levels in three regions (Figure 6) using bisulfite pyrosequencing. The two regions upstream of the transcription start site (TSS) (regions 1 and 2 on Figure 6) were selected as they cover or are directly adjacent to sites that have been shown to be differentially methylated [39], and region 3 (Figure 6) was selected as it spanned the *H19* promoter region and the TSS.

The first region (denoted 1 in Figure 6) encompassed five CpG sites with a mean methylation level of 54.7±7.8%, which would be expected at a differentially methylated imprinted locus. In region 1, there was no significant difference in mean methylation levels between first trimester (54.8±6.9%) and term (53.5±7.2%) placentae or at any of the 5 individual CpG sites (Figure 6, Table S4). The second region assessed (denoted 2 in Figure 6), covered 12 CpG sites which showed overall hypomethylation, with a mean methylation level of 30.9±3.9% in first trimester and 28.9±5.3% in term placentae. When analyzed independently, 4 of the 12 CpG sites, 3 of which are adjacent to each other, showed significantly higher methylation in first trimester placentae in comparison to term placentae (Figure 6). The third region that spanned the *H19* TSS showed mean methylation levels of 16.1±3.1% in first trimester placentae and 15.5±3.5% in term placentae. When each CpG site was analyzed individually, the cytosine nucleotide 39 bp upstream from the *H19* TSS (Figure 6) showed significantly higher methylation (*P* = 0.02) in first trimester placentae (15.2±3.6% vs 12.0±3.2%). Details of DNA methylation levels in first trimester and term placentae at each individual CpG site and the statistical comparisons between the groups are listed in Table S4.

### *H19* Repressed Allele Expression is Correlated with Higher Levels of DNA Methylation

**Figure 7:**
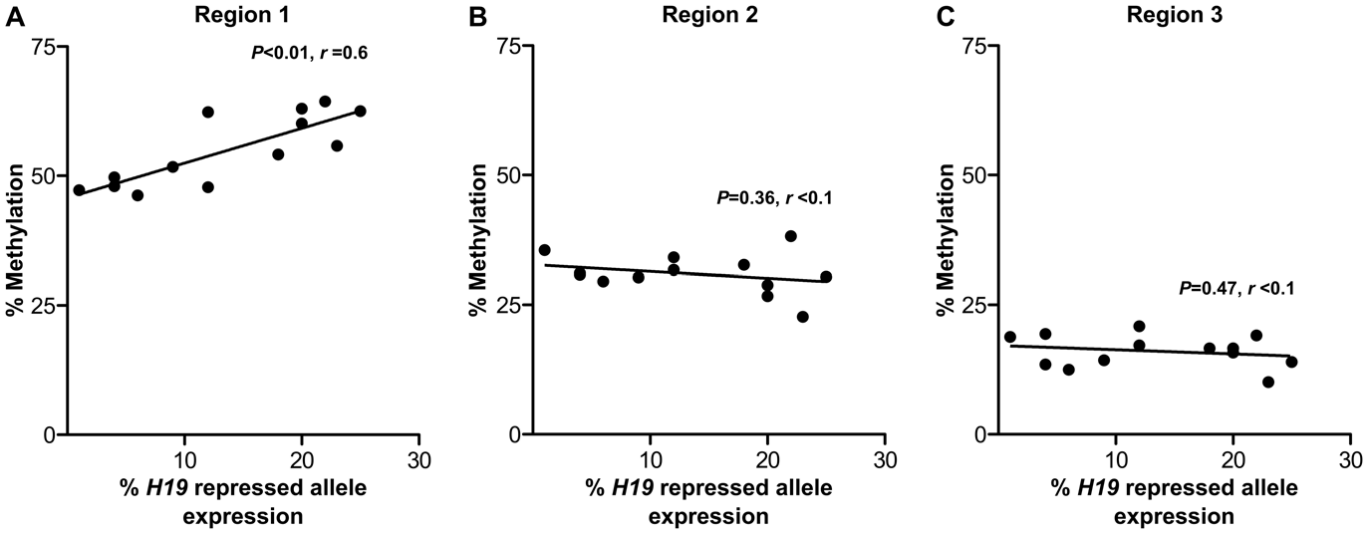
Levels of *H19* repressed allele expression and DNA methylation in human first trimester placentae. (A) Increased expression from the repressed *H19* allele is positively correlated (*P* = 0.0016, r = 0.61) with increased DNA methylation in region 1. (B & C) *H19* repressed allele expression is not correlated with DNA methylation in region 2 (*P* = 0.3626, r = 0.08) or region 3 (*P* = 0.4791, r = 0.04). Each point on the graph represents individual first trimester placenta samples. Methylation levels in each region represent the average methylation from 5 CpG sites in region 1 (Chr11∶2021011–2021070), 12 CpG sites in region 2 (Chr11∶2021011–2021070) and 5 CpG sites in region 3 (Chr11∶2019079–2019145). Genomic coordinates refer to reference assembly GRCh37/hg19.

As distinct variation in expression from the *H19* repressed allele in first trimester placentae was observed, we tested for correlations between the level of repressed allele expression and levels of DNA methylation at CpG’s of first trimester placentae. In region 1, a significant positive correlation (*P*<0.001, r = 0.65) was observed between repressed allele expression and the mean methylation level across the region (Figure 7A). When each of the 5 CpG sites in this region were analyzed independently for the same correlation, the results remained significant for each site (Table S5). This correlation was not observed for region 2 (Figure 7B, *P* = 0.36, r = 0.07) or 3 (Figure 7C, *P* = 0.47, r = 0.05) or for any individual CpG sites within these regions (Table S5).

## Discussion

Imprinted genes are known to be critically involved in placental development and function. Aberrant patterns of imprinted gene expression are implicated in pregnancy complications such as preeclampsia and intrauterine growth restriction [5], [40], [41], [42], [43]. Although the symptoms of these conditions manifest late in pregnancy, their pathogenesis is commonly attributed to compromised first trimester placental development [44]. Previous research on genomic imprinting in the human placenta has focused on the term placenta [25], [32], [36], [41], [45], [46] and data during the first trimester of gestation is limited [27], [34], [35], [47]. In the present study, we investigated the imprinting status (i.e., allele-specific expression) of three genes, *H19*, *IGF2* and *IGF2R*, which have known, but poorly understood, associations with pregnancy complications and placental abnormalities in humans and/or animal models [6], [7], [8], [9], [10]. We assessed allele-specific expression of these genes and DNA methylation in the *H19-IGF2* imprinting control region in first trimester (6–12 weeks of gestation) and term (37–42 weeks of gestation) placentae.

We assessed *IGF2R* allele-specific expression, as the imprinting status of this important gene for prenatal growth and development remains controversial in human. We observed balanced expression from both *IGF2R* alleles, and although we did not investigate any potential imprinting mechanisms for this gene, these results suggest *IGF2R* is not imprinted in the first trimester placenta. Imprinting of *IGF2R* has been suggested to be a polymorphic trait in humans, with a small proportion of individuals showing monoallelic expression or partial imprinting [3], [27], [33]. In this study, we assessed more informative samples than previous studies [3], [27], [33], [48] but found no evidence for polymorphic *IGF2R* imprinting in the placenta. Although we observed overall a balanced expression of alleles for *IGF2R*, individual allelic expression ratios ranged from 36∶64 to 49∶51. This variation may reflect what has been described previously as partial repression or allelic preference [27], [33]. It is presently unclear if this subtle imbalance of *IGF2R* allelic expression is due to genetic variation in allele-specific epigenetic regulation or a parent-of-origin effect.

Allele-specific expression of *H19* showed considerable inter-individual variation, with expression from the repressed (i.e. imprinted) allele contributing up to 25% of total *H19* transcript in the first trimester placenta. In contrast, *IGF2* showed predominantly monoallelic expression and little variation between individuals, with one allele contributing more than 90% of *IGF2* transcript in all investigated samples. This indicated that *IGF2* allele-specific expression is tightly regulated in the first trimester placenta and suggests that *IGF2* imprinting is established early in development and remains stable throughout gestation.

Determining loss of imprinting or biallelic expression of imprinted genes was previously performed by restriction fragment length polymorphism (RFLP) analysis. This method provides a qualitative or semi-quantitative assessment of monoallelic or biallelic expression. In human placentae from uncomplicated pregnancies, *H19* RFLP data showed biallelic expression before 10 weeks of gestation and imprinted expression at term [25], [35]. However, term placentae from preeclamptic pregnancies were reported to display biallelic expression with the RFLP method [25]. This biallelic *H19* expression could indicate a failure to establish correct *H19* imprinting with downstream effects on placental development [25], [35]. The data presented in the current study show that *H19* expression from the imprinted, i.e. repressed, allele can range from 9–22% at 9–10 weeks of gestation, highlighting the potential ambiguity in classifying expression as mono- or biallelic by less sensitive methods. Our data support the view [32], [49] that classification of genes as imprinted or non-imprinted by qualitative methods may be a less meaningful distinction than quantitative measurements of imprinting status based on precise estimates of relative contributions from each allele.

More recently, quantitative PCR and pyrosequencing have been used to evaluate allele-specific expression in placental tissue. By using these highly sensitive methodologies, expression from the “silenced”, imprinted, alleles has been generally higher in first trimester placentae [46] with some variation at term [36]. Both the RFLP assay and quantitative allele-specific expression approaches support the concept that repressed allele expression changes through gestation in the placenta, particularly during early pregnancy [25], [34], [35], [46]. Using placental tissue from 6–12 weeks of gestation, we tested the hypothesis that imprinted allele-specific expression changes during the first trimester of pregnancy. We found no significant differences between early and late first trimester allelic expression ratios for *H19, IGF2* or *IGF2R*. Although we quantified allelic expression ratios using a highly sensitive technique, the method used for classifying gestational age, our sample size, and the proportion of heterozygotes in each group may have prevented the detection of significant changes across first trimester age groups. When comparing first trimester and term placenta samples for *H19,* we found a significant decrease in the proportion of repressed allele expression at term. Furthermore, these results for *H19* show notable inter-individual variation early during placental development, and more uniformity in allelic expression ratios as gestation progresses. This is a clear demonstration of dynamic change in imprinting status well beyond the blastocyst and implantation stages. However, an alternative explanation for the observed differences in *H19* allelic expression ratios between first trimester and term samples in the present study could be the unbiased sampling of material from elective terminations of pregnancy versus the selected material at term that came from normal pregnancies only. Placental tissue from elective terminations of pregnancy in first trimester will, by necessity, include those from pregnancies that may have been destined to develop a pregnancy complication e.g. preeclampsia, preterm labour or intrauterine growth restriction which are typically diagnosed later in gestation. Potentially, first trimester placental samples exhibiting expression from the repressed allele may have been destined to retain biallelic expression and associate with preeclampsia later. However, we consider this unlikely given that 8 out of 13 first trimester samples had greater than 10% expression from the repressed allele and preeclampsia occurs in just 8% of women in the community where our samples were collected [50].

Our results show *H19* expression from the repressed allele is not correlated with expression from the *IGF2* repressed allele in the same samples. The prevailing regulatory model of the *H19-IGF2* region based on differential DNA methylation predicts that both genes are not expressed from a single chromosome. Although this model is supported by considerable evidence [12], [51], [52] (and references cited therein), there is also evidence to suggest that this model may be insufficient (reviewed in [53]). The data presented here show higher expression from the repressed *H19* allele is not correlated with any change in *IGF2* repressed allele expression in individual placentae. Additionally, we show that DNA methylation levels at CpG sites (1946–2005 bp upstream of the *H19* TSS) that flank the 6^th^ CTCF binding domain [39], are positively correlated with the level of expression from the *H19* repressed allele, which was unexpected given the prevailing regulatory model. Furthermore, we observed significantly higher DNA methylation in first trimester placentae in the region 422–524 bp upstream of the *H19* TSS that surrounds the differentially methylated region (DMR) [39], despite finding no correlation with *H19* repressed allele expression. This suggests DNA methylation in the DMR decreases progressively throughout gestation with no effect on *H19* allelic regulation. Together, these findings suggest that the methylation dependant enhancer competition model of the *H19-IGF2* locus may not fully explain the patterns of allele-specific expression observed for these genes in the early human placenta, as suggested previously [53]. However, although we assessed DNA methylation at sites within the *H19*-*IGF2* regulatory region, we did not assess methylation across all the CTCF binding sites upstream of *H19.* Moreover, we did not investigate additional regulatory mechanisms, such as the actions of other non-coding RNA’s and repressive histone modifications that are involved in placental-specific imprinting [54], [55], [56], [57]. Therefore we are unable to rule out other mechanistic changes that may be influencing *H19* allele specific expression.

An important consideration when using placental tissue for studying genomic imprinting is that this organ arises from multiple extra-embryonic and embryonic cell lineages. Cells descended from both the inner cell mass and trophectoderm may show major epigenetic differences [58], and as a result, analysis of whole placental villous tissue may not identify cell lineage-specific imprinting effects. In this study, we show a clear imprinting effect for *IGF2* in all heterozygous first trimester placenta samples, which suggests that all cell types composing the placental villi had *IGF2* imprinting mechanisms in place. However, for *H19* we observed notable inter-individual variation in expression from the imprinted allele. This variation could be due to the heterogeneous nature of the placental villous tissue sampled and *H19* lineage-specific imprinting at the single cell level. Cell-specific imprinted gene expression has been proposed as an *all or none* phenomenon in placental cell lines [59], and *H19* biallelic expression has been shown to be specific to extravillous cytotrophoblast cells [47], suggesting there is no intermediate imprinting effect at the single cell level. Therefore, observing variations in relative expression from the imprinted allele in placental tissue may simply reflect the fraction of cells with complete biallelic expression [59]. As first trimester placental tissue sampling is expected to yield a higher proportion of extravillous cytotrophoblast cells than those collected at term, changes in the level of imprinting across gestation may reflect proportional changes in cell lineage populations as the placenta differentiates. This suggests future studies of placental imprinting dynamics should consider the potential influence of placental cell type heterogeneity.

The *H19, IGF2* and *IGF2R* genes have key roles in placental development, yet the phenotypic effect of their allele-specific expression across gestation remains unknown. However, the role of *H19* as a regulator of the recently described imprinted gene network suggests potentially significant phenotypic effects [15]. This may depend on differences in gene dosage, but could also involve more complex regulatory effects in *trans*. To date, the normal developmental patterns of imprinted gene expression in the human placenta are poorly understood. As altered patterns of imprinting in term placentae are associated with pregnancy complications, identifying when these abnormal patterns are established may aid in elucidating the origins of placental abnormalities implicated in their aetiology. Our results highlight the requirement for robust and sensitive methods to determine the role of imprinted allele-specific expression in placentae from complicated pregnancies. Undoubtedly, precise methods and comprehensive studies will be required to progress towards understanding the molecular basis of potentially life threatening pregnancy complications in which defective placentation is implicated.

## Supporting Information

Table S1 **Genomic DNA specific primers used to detect DNA contamination in RNA samples.**
(PDF)

Table S2 **Details of genes, SNP regions and primers used for quantifying allele-specific expression by pyrosequencing.**
(PDF)

Table S3 **Parental and placental genotypes with placental allele expression ratio for** ***H19*** **and** ***IGF2*** **.**
(PDF)

Table S4 **Comparison of DNA methylation levels at individual CpG loci in 3 regions upstream and surrounding the** ***H19*** **transcription start site in human first trimester and term placentae.**
(PDF)

Table S5 **Pearson’s correlation of** ***H19*** **repressed allele expression and DNA methylation levels at individual CpG loci in human first trimester placentae.**
(PDF)

Methods S1 **Genotyping single nucleotide polymorphisms by PCR and High Resolution Melt (HRM) analysis.**
(PDF)
