# Supplementary material for: Quantitative Allele-Specific Expression and DNA Methylation Analysis of H19, IGF2 and IGF2R in the Human Placenta across Gestation Reveals H19 Imprinting Plasticity
Source: PLoS One. 2012 Dec 5;7(12):e51210. doi: 10.1371/journal.pone.0051210 (PMC3515552; doi:10.1371/journal.pone.0051210)
Supplement: Table S1 — Genomic DNA specific primers used to detect DNA contamination in RNA samples. (PDF) [file pone.0051210.s001.pdf]

Table S2. Genomic DNA specific primers used to detect gDNA contamination in RNA samples.

Gene	Location	Direction	Primer Sequence (5'-3')
IGF2R	Intron	Fwd	GCCTCTTCTTGTTAATTTCCCTGTT
	Exon	Rev	TTCAGTTTCTCCACAGACATTCAA
