# Supplementary material for: Quantitative Allele-Specific Expression and DNA Methylation Analysis of H19, IGF2 and IGF2R in the Human Placenta across Gestation Reveals H19 Imprinting Plasticity
Source: PLoS One. 2012 Dec 5;7(12):e51210. doi: 10.1371/journal.pone.0051210 (PMC3515552; doi:10.1371/journal.pone.0051210)
Supplement: Table S2 — Details of genes, SNP regions and primers used for quantifying allele-specific expression by pyrosequencing. (PDF) [file pone.0051210.s002.pdf]

Table S3. Details of genes, SNP regions and primers used for quantifying allele-specific expression by pyrosequencing.

Gene	SNP	PCR primer sequence (5'-3')	Amplicon size (bp)	Pyrosequencing primer (5'-3')
H19	rs217727	Fwd-CGGCGACTCCATCTTCATG	75	ATGGCCACCCCCTGCG
		Rev-(B)TCCAGCTCTGGGATGATGTG		
IGF2	rs680	Fwd-TGGCCAGTTTACCCTGAAAATTC	116	CCTGTGATTTCTGGG
		Rev-(B)TGGACTTGAGTCCCTGAACCA		
IGF2R	rs998075	Fwd-CTCGGTGTGTGTCTTTCATTGTT	73	TGTCTTTCATTGTTATAGGG
		Rev-(B)CATATTATGATGGGATGATCCAAC		
IGF2R	rs1570070	Fwd-AGCAGCAGGATGTCTCCATAG	118	CCCAGAGCGGAGGTT
		Rev-(B)TGTATTTCAGTTTCTCCACAGACA		

(B) denotes 5' nucleotide biotin modification
