# Supplementary material for: Quantitative Allele-Specific Expression and DNA Methylation Analysis of H19, IGF2 and IGF2R in the Human Placenta across Gestation Reveals H19 Imprinting Plasticity
Source: PLoS One. 2012 Dec 5;7(12):e51210. doi: 10.1371/journal.pone.0051210 (PMC3515552; doi:10.1371/journal.pone.0051210)
Supplement: Table S3 — Parental and placental genotypes with placental allele expression ratio for H19 and IGF2 . (PDF) [file pone.0051210.s003.pdf]

Table S4. Parental and placental genotypes with placental allele expression ratio for *H19* and *IGF2*.

	***H19* rs217727 genotypes**			**Placenta allele expression ratio**		
**Sample**	**Maternal**	**Paternal**	**Placenta**	**C**	**T**	**Expression**
41	C/T	-	C/T	0.02	0.98	ND
42	C/T	C	C/T	0.01	0.99	Maternal
43	T/T	-	C/T	0.04	0.96	Maternal
45	C/T	-	C/T	0.05	0.95	ND
47	C/C	C/T	C/T	0.97	0.03	Maternal
48	C/T	C	C/T	0.01	0.99	Maternal
49	C/T	C	C/T	0.03	0.97	Maternal
51	C/C	C/T	C/T	0.98	0.02	Maternal
52	C/C	C/T	C/T	0.95	0.05	Maternal
53	C/T	C	C/T	0.02	0.99	Maternal
55	C/C	C/T	C/T	0.91	0.09	Maternal
59	C/T	-	C/T	0.03	0.97	ND
61	C/T	C/T	C/T	0.04	0.96	ND
68	C/C	-	C/T	0.97	0.03	Maternal
69	C/T	C/T	C/T	0.02	0.98	Maternal

	***IGF2* rs680 Genotypes**			**Placenta allele expression ratio**		
**Sample**	**Maternal**	**Paternal**	**Placenta**	**C**	**T**	**Expression**
40	C	C/T	C/T	0.01	0.99	Paternal
41	C	-	C/T	0.02	0.99	Paternal
44	C/T	C/T	C/T	0.06	0.94	ND
46	-	C/T	C/T	0.01	0.99	ND
47	C/T	C/T	C/T	0.02	0.98	ND
50	C/T	-	C/T	0.02	0.98	ND
51	C/T	C/T	C/T	0.02	0.98	ND
52	C	-	C/T	0.01	0.99	Paternal
54	C/T	C/T	C/T	0.99	0.01	ND
56	T	C/T	C/T	0.99	0.01	Paternal
57	C/T	-	C/T	0.98	0.02	ND
58	T	C	C/T	0.99	0.02	Paternal
60	C	C/T	C/T	0.01	1.00	Paternal
61	C/T	C/T	C/T	0.02	0.98	ND
62	-	C/T	C/T	0.98	0.02	ND
63	T	C	C/T	0.98	0.02	Paternal
66	C/T	C/T	C/T	0.02	0.98	ND
67	T	-	C/T	0.99	0.02	Paternal
68	T	-	C/T	0.98	0.02	Paternal

ND = Could not be determined
