# Supplementary material for: Quantitative Allele-Specific Expression and DNA Methylation Analysis of H19, IGF2 and IGF2R in the Human Placenta across Gestation Reveals H19 Imprinting Plasticity
Source: PLoS One. 2012 Dec 5;7(12):e51210. doi: 10.1371/journal.pone.0051210 (PMC3515552; doi:10.1371/journal.pone.0051210)
Supplement: Table S4 — Comparison of DNA methylation levels at individual CpG loci in 3 regions upstream and surrounding the H19 transcription start site in human first trimester and term placentae. (PDF) [file pone.0051210.s004.pdf]

Table S5. Comparison of DNA methylation levels at individual CpG loci in 3 regions upstream and surrounding the *H19* transcription start site (TSS) in human first trimester and term placentae.

	**Distance (bp) from TSS**	**Genomic location (GRCh37/hg19)**	**% DNA Methylation**		**P value**
			**First trimester N=13 Mean±SD**	**Term N=18 Mean±SD**	
**Region 1**	-2004	2021069	47.0±7.5	45.6±7.1	0.5899
	-1986	2021051	46.5±6.2	45.1±6.3	0.5527
	-1967	2021032	50.1±6.8	49.7±6.6	0.8717
	-1959	2021024	59.9±7.9	57.6±8.9	0.4636
	-1947	2021012	70.6±7.2	69.4±9.3	0.7001
	Region mean		54.8±6.9	53.5±7.2	0.6065
**Region 2**	-603	2019668	38.9±6.0	39.3±7.7	0.8885
	-591	2019656	35.9±4.3	36.5±6.5	0.7559
	-569	2019634	32.4±2.5	32.2±4.7	0.8808
	-562	2019627	34.5±5.8	36.0±7.0	0.5248
	-560	2019625	31.7±4.5	32.0±5.9	0.8789
	-542	2019607	27.2±8.1	23.5±10.2	0.2959
	**-523**	**2019588**	**38.7±4.4**	**32.7±6.2**	**0.0056**
	-504	2019569	31.4±6.6	33.8±7.4	0.3604
	-502	2019567	32.5±4.3	30.0±6.4	0.2361
	**-484**	**2019549**	**32.5±4.2**	**23.7±4.6**	**< 0.0001**
	**-437**	**2019502**	**19.5±2.6**	**14.8±2.2**	**< 0.0001**
	**-423**	**2019488**	**16.2±2.4**	**11.7±2.8**	**< 0.0001**
	Region mean		30.9±3.9	28.9±5.3	0.2377
**Region 3**	**-39**	**2019144**	**15.2±3.6**	**12.0±3.2**	**0.0152**
	-25	2019130	24.7±5.5	27.9±4.8	0.0927
	-12	2019117	21.6±4.6	21.1±4.3	0.7552
	+15	2019091	10.0±1.9	8.8±3.2	0.2427
	+26	2019080	8.8±2.9	9.9±4.2	0.4347
	Region mean		16.1±3.1	15.5±3.5	0.6467
