# Supplementary material for: Quantitative Allele-Specific Expression and DNA Methylation Analysis of H19, IGF2 and IGF2R in the Human Placenta across Gestation Reveals H19 Imprinting Plasticity
Source: PLoS One. 2012 Dec 5;7(12):e51210. doi: 10.1371/journal.pone.0051210 (PMC3515552; doi:10.1371/journal.pone.0051210)
Supplement: Table S5 — Pearson’s correlation of H19 repressed allele expression and DNA methylation levels at individual CpG loci in human first trimester placentae. (PDF) [file pone.0051210.s005.pdf]

Table S6. Pearson's correlation of *H19* repressed allele expression and DNA methylation levels at individual CpG loci in human first trimester placentae.

	Distance (bp) from TSS	Genomic location (GRCh37/hg19)	P value	R^2 value
**Region 1**	**-2004**	**2021069**	**0.0011**	**0.6348**
	**-1986**	**2021051**	**0.0008**	**0.6540**
	**-1967**	**2021032**	**0.0017**	**0.6062**
	**-1959**	**2021024**	**0.0007**	**0.6630**
	**-1947**	**2021012**	**0.0077**	**0.4898**
	**Region mean**		**0.0008**	**0.6527**
**Region 2**	-603	2019668	0.1255	0.1999
	-591	2019656	0.2814	0.1045
	-569	2019634	0.3068	0.0945
	-562	2019627	0.1494	0.1793
	-560	2019625	0.2429	0.1216
	-542	2019607	0.7337	0.0110
	-523	2019588	0.6850	0.0155
	-504	2019569	0.5050	0.0414
	-502	2019567	0.5199	0.0386
	-484	2019549	0.4551	0.0517
	-437	2019502	0.3780	0.0712
	-423	2019488	0.4514	0.0525
	Region mean		0.3626	0.0758
**Region 3**	-39	2019144	0.7795	0.0074
	-25	2019130	0.2029	0.1429
	-12	2019117	0.3004	0.0969
	+15	2019091	0.1589	0.1719
	+26	2019080	0.6730	0.0168
	Region mean		0.4791	0.0465
