# Supplementary material for: Quantitative Allele-Specific Expression and DNA Methylation Analysis of H19, IGF2 and IGF2R in the Human Placenta across Gestation Reveals H19 Imprinting Plasticity
Source: PLoS One. 2012 Dec 5;7(12):e51210. doi: 10.1371/journal.pone.0051210 (PMC3515552; doi:10.1371/journal.pone.0051210)
Supplement: Methods S1 — Genotyping single nucleotide polymorphisms by PCR and High Resolution Melt (HRM) analysis. (PDF) [file pone.0051210.s006.pdf]

Methods S1

DNA Genotyping by HRM

First trimester placenta DNA was genotyped for *IGF2* and *IGF2R* SNPs by PCR and high-resolution melt (HRM) analysis. PCR primers (Table 1) were custom designed using Primer Express (v2.0 Applied Biosystems), with amplicon melting characteristics assessed using DinaMelt [1] and checked for specificity using the UCSC *in silico* PCR tool (http://genome.ucsc.edu) with the GRCh37/hg19 reference genome [2,3]. All HRM oligonucleotides were manufactured by GeneWorks (Adelaide). Gene, primer and amplicon details for the PCR-HRM reactions are listed in Table 1. PCR-HRM was performed in 10µL reactions, including 25ng of genomic DNA, 250nM of each forward and reverse primer, 5µL of either SsoFast EvaGreen Supermix (Bio-Rad) for *IGF2R* rs1570070 or MeltDoctor (Applied Biosystems) for *IGF2R* rs998075 and *IGF2* rs680 using the Corbett Rotor-Gene 6000. Initial denaturation was 98˚C for 2 minutes (SsoFast) or 10 minutes (MeltDoctor), followed by 40-45 cycles of 2-step temperature cycling of 98˚C for 5 seconds and 60˚C for 20 seconds.

Immediately following PCR, samples were held at 50˚C for 30 seconds before temperature ramping at 0.1˚C steps at 2 seconds per step across a melt domain of >10˚C, which was specific for each amplicon (Table 1). HRM curves were analysed using Corbett Rotor-Gene software (Corbett Research version 1.7, build 87). Normalisation was performed using windows of 0.5˚C at least 2˚C before the first melt transition and at least 1˚C after the sample had completely melted. Genotypes were called at 90% confidence by comparison to sequence verified controls using the Corbett Research HRM Software v1.7 (Figure 1A). Control genotypes were confirmed by visually checking sequence chromatograms (Figure 1B) generated by the ABI 3130xl genetic analyser at Flinders and SouthPath Sequencing Facility, South Australia.

Table 1. Gene, SNP, and HRM genotyping assay details used for genotyping placental DNA.

Gene	SNP	PCR primer sequence (5'-3')	Amplicon size (bp)	HRM range (˚C)
IGF2	rs680	Fwd-TGGCCAGTTTACCCTGAAAATTC	116	75-88
		Rev-TGGACTTGAGTCCCTGAACCA		
IGF2R	rs1570070	Fwd-GCCTCTTCTTGTTAATTTCCCTGTT	95	67-77
		Rev-TTCAGTTTCTCCACAGACATTCAA		
IGF2R	rs998075	Fwd-CTCGGTGTGTGTCTTTCATTGTT	73	69-81
		Rev-CATATTATGATGGGATGATCCAAC		

SNP = Single Nucleotide Polymorphism, HRM = High Resolution Mel

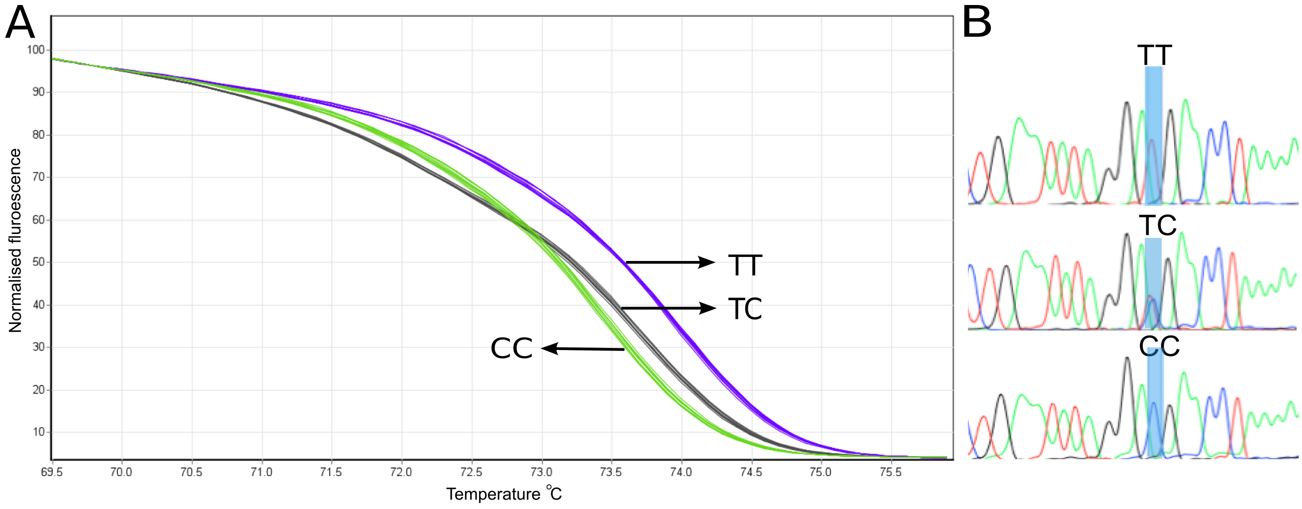

Figure 1. Genotyping of *IGF2R* rs1570070 by HRM with sequenced controls.

A Normalised HRM melt plot showing rs1570070 TT, TC and CC genotypes. Samples are grouped into distinct genotype curves. Green and purple curves represent homozygous samples and grey curve represents heterozygous samples. Each curve group includes a sequenced control sample.

B Chromatogram traces confirm control sample genotypes, polymorphic site highlighted for each genotype.

References

1. Markham NR, Zuker M (2005) DINAMelt web server for nucleic acid melting prediction. Nucleic Acids Research 33: W577.
2. Kuhn RM, Karolchik D, Zweig AS, Wang T, Smith KE, et al. (2009) The UCSC genome browser database: update 2009. Nucleic Acids Research 37: D755.
3. Kent WJ (2002) BLAT: the BLAST-like alignment tool. Genome Research 12: 656.
